# Therapeutic advances in the treatment of vasculitis

**DOI:** 10.1186/s12969-016-0082-8

**Published:** 2016-04-26

**Authors:** Despina Eleftheriou, Paul A. Brogan

**Affiliations:** ARUK centre for Paediatric and Adolescent Rheumatology, Institute of Child Health and Great Ormond St Hospital NHS Foundation Trust, 30 Guilford Street, London, WC1N 1EH UK; Department of Paediatric Rheumatology, Institute of Child Health and Great Ormond St Hospital NHS Foundation Trust, 30 Guilford Street, London, WC1 E1N UK

## Abstract

Considerable therapeutic advances for the treatment of vasculitis of the young have been made in the past 10 years, including the development of outcome measures that facilitate clinical trial design. Notably, these include: a recognition that some patients with Kawasaki Disease require corticosteroids as primary treatment combined with IVIG; implementation of rare disease trial design for polyarteritis nodosa to deliver the first randomised controlled trial for children; first clinical trials involving children for anti-neutrophil cytoplasmic antibody (ANCA) vasculitis; and identification of monogenic forms of vasculitis that provide an understanding of pathogenesis, thus facilitating more targeted treatment. Robust randomised controlled trials for Henoch Schönlein Purpura nephritis and Takayasu arteritis are needed; there is also an over-arching need for trials examining new agents that facilitate corticosteroid sparing, of particular importance in the paediatric population since glucocorticoid toxicity is a major concern.

## Background

Primary systemic vasculitides of the young are relatively rare diseases, but are associated with significant morbidity and mortality, particularly if there is diagnostic delay [1, 2]. There have been a number of notable advances recently in the field of paediatric vasculitis research, including the development of classification criteria; and tools to assess disease outcome for use in clinical trials and other research involving children with vasculitis [3, 4]. Treatment regimens continue to improve, with the use of different immunosuppressive medications and newer therapeutic approaches such as biologic agents [5–7]. With the exception of Kawasaki Disease, most of current treatment approaches for paediatric vasculitides are based on evidence from small case series, anecdotal observations, or adult studies [5–7]. Therefore, treatment differs substantially throughout Europe; and even within a single country, experience and practices vary considerably. Given that vasculitides are rare, conducting large randomised controlled trials using traditional clinical trial design is usually not feasible. There is, however, a need for a standardized approach to the management of these rare paediatric rheumatic diseases [8]. The SHARE (Single-Hub Access for Pediatric Rheumatology in Europe [8]) project has been set up to address this unmet need, and one of the main aims is to provide recommendations for the management of paediatric rheumatological diseases in European countries. Within this context the SHARE vasculitis working group have recently provided consensus and (where possible) evidence-based statements to optimize and harmonize management of vasculitis (manuscript in preparation). A detailed description of this as yet unpublished guidance is beyond the scope of this review. It is also currently unclear how the impact of the SHARE guidance will be assessed for all the vasculitides; comparative effectiveness research methodologies may be required within the context of national or international patient registries. Indeed this approach is already underway for Kawasaki disease (KD), in the context of a British Paediatric Surveillance Unit study specifically examining the impact of new clinical guidelines for KD in the UK.

This review summarises therapeutic advances in current management strategies for systemic vasculitides, and describes important unmet needs. At the time of writing this review, the SHARE guidelines are not yet published; however, we have aligned the descriptions of vasculitis treatment with the SHARE recommendations, which the reader should refer to (when these become available) for more detailed guidance.

## HSP IgA vasculitis (Henoch–Schönlein purpura)

IgA vasculitis is the new term for Henoch-Schönlein purpura (HSP), and is the commonest systemic vasculitis in childhood [9]. For the purposes of this review, however, we will use the term HSP since that is the term used in the paediatric classification criteria [4], and the one most paediatricians are familiar with. It is defined in the latest Chapel Hill nomenclature (2012) as: vasculitis with IgA1-dominant immune deposits affecting small vessels (predominantly capillaries, venules, or arterioles); often involving skin, gastrointestinal tract and frequently causes arthritis; and associated with glomerulonephritis which is indistinguishable from IgA nephropathy [10]. Classification criteria (Ankara 2008) are: palpable purpura in a predominantly lower limb distribution with at least 1 of 4 of: diffuse abdominal pain; any biopsy showing IgA deposition (mandatory criterion if rash is atypical); arthritis and/or arthralgia; haematuria and/or proteinuria [4]. HSP has a variable and often relapsing course without specific laboratory findings, with a third of children having symptoms up to two weeks; another third up to 1 month; and recurrence of symptoms within 4 months of resolution in the remaining third [11]. Henoch–Schönlein nephritis (HSN) accounts for 1.6–3 % of all childhood cases of end-stage renal failure (ESRF) in the UK [12]. Generally speaking, HSP is a more severe illness in adults than in children [13].

### Therapeutic advances

There is a very poor evidence base to guide the management of HSP, particularly for those with the severe forms of HSP nephritis (HSPN), and thus very few true therapeutic advances. Early morbidity in the disease is due to GI involvement; late morbidity and the most important overall determinant of poor outcome is renal involvement [11]. In children the management of HSP is mainly conservative because the extra renal manifestations are usually self-limited [14]. Arthritis responds well to non-steroidal anti-inflammatory drugs (NSAIDs) [14]. Severe skin lesions and gastrointestinal involvement could require a short course of an oral corticosteroid [14]. Controlled studies have shown that corticosteroids do not prevent renal disease [15, 16]. Despite that, patients with severe renal involvement usually do require corticosteroids combined with other immunosuppressive agents, and sometimes anti-proteinuric and antihypertensive agents [13].

### Summary of the SHARE guidelines for HSP

The SHARE initiative represents an important major therapeutic contribution since it provides consensus guidance for the management of HSP and HSPN, amongst other vasculitides [8]. In anticipation that these will be published imminently, a brief overview of the SHARE management algorithm for HSPN is as follows. Children with microscopic haematuria without renal dysfunction or proteinuria, and those with non-persistent mild-moderate proteinuria usually do not require any specific therapeutic intervention other than a “watchful waiting approach” since the prognosis is excellent. Those with more severe proteinuria and/or impaired glomerular filtration, and those with persistent proteinuria should be reviewed by a paediatric nephrologist, and a renal biopsy is usually recommended. Treatment thereafter includes first line therapy with corticosteroids: oral for all; and initially intravenous pulsed methylprednisolone for those with more severe renal involvement. For the severest cases, intravenous cyclophosphamide is usually required (sometimes with plasma exchange: seek expert advice) as additional first line treatment combined with corticosteroids. Immunosuppressants including azathioprine, mycophenolate mofetil (MMF), or intravenous cyclophosphamide may be considered as second-line agents for those with moderate HSPN. Whilst awaiting publication of the SHARE guidelines, for a comprehensive review of the treatment of HSPN, the reader is referred to [17]. It must be emphasised, however, that in the absence of robust data for evidence supporting the treatment of nephritis, even in view of the much anticipated SHARE guidance, a randomised controlled trial for the treatment of HSPN is urgently needed.

## ANCA associated vasculitis (AAV)

Antineutrophil cytoplasmic autoantibody (ANCA)-associated vasculitides (AAV) comprise granulomatosis with polyangiitis (GPA; previously referred to as Wegener’s granulomatosis), microscopic polyangiitis (MPA), eosinophilic granulomatous polyangiitis (EGPA; previously referred to as Churg-Strauss syndrome), and the so-called renal-limited vasculitis [13]. From a clinical perspective it may be useful to think of GPA as having two forms: a predominantly granulomatous form with mainly localised disease with chronic course; and a florid, acute small vessel vasculitic form characterised by severe pulmonary haemorrhage and/or rapidly progressive vasculitis, or other severe vasculitic manifestation [13]. These two broad presentations may co-exist or present sequentially in individual paediatric and adult patients [13].

### Therapeutic advances

Renal morbidity and mortality is a major concern in the AAV, hence therapy aimed at preservation of renal function is a recurring theme for the treatment of AAV in both adults and children [18]. Treatment for paediatric AAV is broadly similar to the approach in adults based on evidence derived from a number of clinical trials conducted by the European Vasculitis Study group (EUVAS) [19]. The EUVAS group was developed to conduct interventional clinical trials aimed at optimizing treatments and outcomes for vasculitis patients [19]. A total of 12 clinical trials involving over 1300 patients have been conducted that have defined the standard of care and permitted consensus treatment recommendations [19]. Particular areas of focus were: the reduction in cyclophosphamide exposure; efficacy of plasma exchange in reducing renal morbidity; remission maintenance strategies; and establishing the efficacy of newer therapies [19]. Notably, two of the clinical trials have involved children with AAV. These clinical trials, and how they have informed current practice for both adults and children with AAV are summarised below.

The CYCAZAREM trial (CYClophosphamide or AZAthioprine for REMission) recruited 144 new adult patients with AAV and compared a 3–6-month cyclophosphamide course, stopping when remission was achieved, to a standard 12-month course of cyclophosphamide. Both groups were then switched to azathioprine as maintenance therapy [20]. No differences in remission or relapse rates were observed [20]. The validation of sequential cyclophosphamide induction followed by azathioprine maintenance has subsequently served as the basis for later trials [20]. Pulsed IV cyclophosphamide (CYCLOPS, CYCLophosphamide Oral versus PulSed) was then shown to be as effective as daily oral cyclophosphamide, with a 50 % reduction in cumulative cyclophosphamide dose, and fewer adverse events [21]. Based on these studies, current induction of remission therapy in adults and children with AAV includes corticosteroids and cyclophosphamide (usually 6–10 intravenous doses at 500–1000 mg/m2 [maximum 1.2 g] per dose, given every 3–4 weeks) [13, 19]. Intravenous pulsed cyclophosphamide is increasingly favoured over oral continuous cyclophosphamide in adults and children, because the reduced cumulative dose, and less neutropenic sepsis in adult patients, albeit without good prospective paediatric evidence [13, 19].

In addition, B-cell depletion with rituximab has been explored as an alternative to cyclophosphamide for remission induction for AAV [22, 23]. Efficacy of this treatment approach for severe renal disease in combination with two IV cyclophosphamide doses was evaluated in the RITUXVAS trial [23]. This regimen had no benefit over a standard IV cyclophosphamide regimen in terms of efficacy or adverse event rate; but demonstrated that rituximab could provide clinically important cyclophosphamide sparing [23]. Another US multicentre, randomized trial compared rituximab with cyclophosphamide for remission induction, and showed that rituximab was not inferior to daily oral cyclophosphamide for induction of remission in severe ANCA-associated vasculitis; and rituximab may be superior in patients with relapsing disease [22]. These studies led to licensing of rituximab for remission induction of AAV in adults in the USA and Europe. Whether low-dose cyclophosphamide still has a role in induction therapies alongside rituximab remains uncertain. Notably, an ongoing international multicentre study is exploring the efficacy and safety of Rituximab in children with new onset, or relapsing AAV (the PEPRS study; NCT01750697). Whilst we are encouraged that industry are supporting and undertaking paediatric studies of new therapeutic agents in rare diseases such as AAV, we appreciate that studies of “older” drugs, or studies of combination drug therapy in the paediatric population may not be feasible (due to lack of industry support), even if desirable for some diseases.

Two further EUVAS trials evaluated whether cyclophosphamide could be replaced as an induction agent, either with methotrexate or MMF. The NORAM trial (Non-Renal Alternative treatment with Methotrexate) demonstrated that remission rates for non-severe GPA/MPA were similar at 6 months between an oral methotrexate and an oral cyclophosphamide regimen; but that late relapse was more common after methotrexate [24]. MMF also proved not inferior to an IV cyclophosphamide regimen at 6 months in the MYCophenolate mofetil versus CYClophosphamide; MYCYC), trial but with a higher relapse rate in the MMF group. The higher relapse rate was confined to the PR3-ANCA-positive subgroup, and no differences in remission or relapse rates were seen in the MPO-ANCA patient subgroup [25]. Importantly, for the first time, children were included in the MYCYC trial [25]. Many differences exist in physiology, pathology, pharmacokinetics, and pharmacodynamics between children and adults. Inclusion of paediatric patients in large adult RCTs, therefore, by no means eliminates the need for separate paediatric studies, but this approach at least allows us to obtain some robust paediatric data rather than just directly extrapolating from purely adult studies.

Regarding maintenance regimens, the EUVAS group found MMF not to be superior to azathioprine for relapse prevention of AAV after cyclophosphamide induction in the IMPROVE trial [26]. MMF is therefore not recommended as a routine remission maintenance agent, but can be used when azathioprine or methotrexate have failed to maintain remission in adults and children with AAV.

Rituximab was also considered to have role in relapse prevention, and repeat-dose rituximab was shown to be associated with fewer relapses than azathioprine, following cyclophosphamide induction in the French MAINRITSAN trial [27]. An ongoing study led by both EUVAS and the Vasculitis Clinical Research Consortium (VCRC) is exploring the role of rituximab in treatment of relapsing disease in the RITAZAREM trial (NCT01697267).

Other biologic agents that have been considered in AAV therapy are anti-tumour necrosis alpha agents, but these were largely abandoned after a negative result in relapse prevention in an etanercept study [28]. The T-cell co-stimulation inhibitor CTLA4-Ig (abatacept) is currently being studied in a randomized trial for treatment of non-severe, relapsing GPA (ABROGATE; NCT02108860). The ALEVIATE trial (NCT01405807), a small dose-finding trial of compassionate use of alemtuzumab (a monoclonal antibody against CD-52, found on mature lymphocytes), is ongoing. For EGPA, a single-centre, phase 2, uncontrolled study demonstrated that mepolizumab (a monoclonal antibody against IL-5) allowed glucocorticoid sparing over the course of the disease in most patients, with no relapses during the active 9-month treatment phase [29].

Lastly, the PEXIVAS trial is comparing four different therapeutic combinations in a factorial design examining a reduced oral glucocorticoid regimen with a standard-dose regimen; with or without plasma exchange for adults with AAV and renal involvement (NCT00987389). Small case series have also indicated that plasma exchange reduces renal morbidity in children with AAV [30]; but there are no ongoing or planned trials of therapeutic plasma exchange in children with AAV.

## Kawasaki disease (KD)

Kawasaki disease (KD) is a self-limiting vasculitic syndrome that predominantly affects medium and small-sized arteries [31]. KD has a worldwide distribution with a male preponderance, an ethnic bias towards oriental children, some seasonality, and occasional epidemics [31]. The principal clinical features of Kawasaki disease are: (i) Fever persisting for ≥5 days; (ii) Peripheral extremity changes (reddening of the palms and soles, indurative oedema and subsequent desquamation); (iii) Polymorphous exanthema; (iv) bilateral conjunctival injection/congestion; (v) lips and oral cavity changes (reddening/cracking of lips, strawberry tongue, oral and pharyngeal injection); (vi) acute non-purulent cervical lymphadenopathy [32]. For the diagnosis of Kawasaki disease to be formally established five of the above six clinical features should be present [32]. Children with fewer than five of the six principal features can be diagnosed with Kawasaki disease when coronary aneurysm or dilatation is recognized by two-dimensional echocardiography or coronary angiography [32].

### Therapeutic advances

Early recognition and treatment of KD with aspirin and intravenous immunoglobulin (IVIG) has been shown unequivocally by randomised controlled trials and meta-analysis to reduce the occurrence of CAA [32, 33]. Currently, aspirin at a dose of 30–50 mg/kg/day is recommended during the acute phase of the illness, as this may be better tolerated than higher doses in terms of gastrointestinal and other side effects [31, 32, 34]. The dose should be reduced to an anti-platelet does of 3–5 mg/Kg once fever and inflammation have subsided [31, 32, 34]. Notably however, IVIG resistance has been reported in up to 20 % of cases and these patients are at increased risk of developing CAA unless they receive additional treatment [31]. Corticosteroids are effective treatment for other forms of vasculitis, but early retrospective analyses suggested that corticosteroids were associated with increased risk of CAA [31]. However, this almost certainly reflected selection bias as the sickest patients received steroids. Importantly, clinical trials evaluating the use of corticosteroids plus IVIG have produced seemingly confusing results, exemplified by a USA study of high dose methyl prednisolone showing no reduction in CAA, and the recent Japanese RAISE study (using a lower dose but longer duration of prednisolone) showing improved outcome [6, 35, 36]. Chen et al. recently reported a meta-analysis comparing the frequency of CAA in patients treated with IVIG plus corticosteroids or IVIG alone for the primary treatment of KD [37]. They found that significantly fewer patients receiving IVIG + corticosteroids developed CAA than those receiving IVIG alone (7.6 % versus 18.9 %; OR: 0.3; 95 % CI 0.20–0.46); there was no significant differences in frequency of severe adverse events between the steroid and non-steroid treatment groups [37]. Chen’s meta-analysis provides convincing evidence that steroids combined with IVIG as initial treatment reduces overall risk of CAA in severe KD [37]. However, neither the meta-analysis nor the RAISE study provides clear answers as to whether all children should be treated with corticosteroids, and what dose, duration and route of corticosteroids should be used [36, 37].

**Fig. 1:**
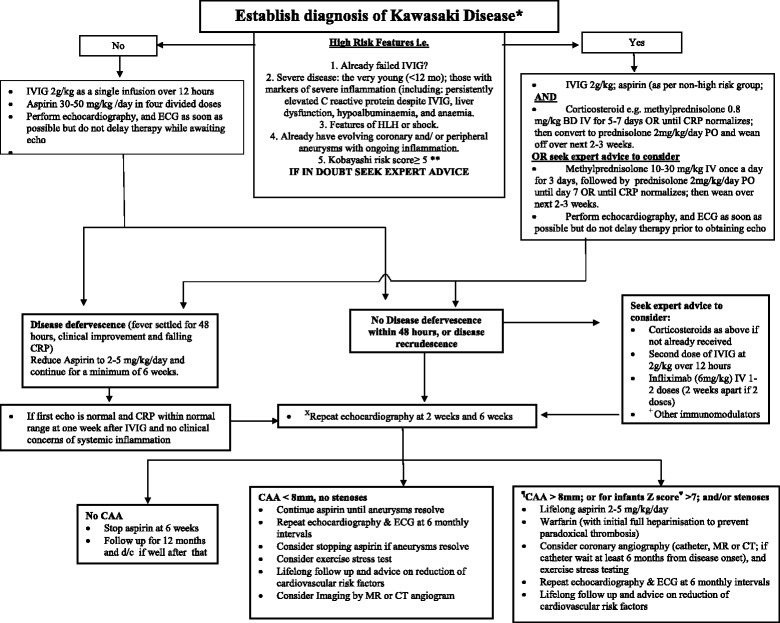
Recommended clinical guideline for the management of Kawasaki disease. Adapted from reference 31. *Treatment can be commenced before 5 days of fever if sepsis excluded; treatment should also be given if the presentation is > 10 days from fever onset if there are signs of persistent inflammation; **Kobayashi risk score ≥5 points X Refer to paediatric cardiologist; ¶ Other specific interventions such as PET scanning, addition of calcium channel blocker therapy, and coronary angioplasty at discretion of paediatric cardiologist. + Other immunomodulators may include ciclosporin. ♥For infants, Z score for internal coronary artery diameter >7 based on Montreal normative http://parameterz.blogspot.co.uk/2010/11/montreal-coronary-artery-z-scores.html

With these caveats in mind, we recently proposed a pragmatic treatment approach for the use of corticosteroids based on the current data and recognise that some children would benefit from combination therapy of corticosteroid and IVIG as first line therapy for KD (Fig. 1 [31].)

Furthermore, there are emerging animal data and case reports suggesting a role for anti-TNF-α for the treatment of KD [7, 38, 39]. The most commonly used agent is infliximab, a chimeric murine/human IgG1 monoclonal antibody specifically binding TNF-α [7, 38, 39]. A recent multicentre study in the USA comparing IVIG and aspirin to IVIG/aspirin plus infliximab as initial therapy in an unselected group of KD patients has been completed and showed no reduction in CAA, although there was faster resolution of the acute phase response in the infliximab group [7]. Although there was a trend towards reduced CAA injury, the study was underpowered to detect significant reduction in CAA [7]. Thus, anti-TNFα should be considered in patients with IVIG resistant KD, but not routinely as adjunctive and further study is required of its role in initial therapy primary therapy.

A number of other therapies are also currently explored in KD. An open label study is currently examining the efficacy of anakinra as first line therapy in KD (NCT02390596; KAWAKINRA). Another phase III multicentre, randomised, open-label, blinded-end point trial is currently evaluating the efficacy and safety of immunoglobulin plus ciclosporin in patients with severe KD in Japan (the KAICA Trial).

## Polyarteritis nodosa (PAN)

Polyarteritis nodosa (PAN) is a primary systemic necrotizing vasculitis predominantly targeting medium-sized arteries [1]. The classification criteria for childhood PAN are histologic evidence of necrotizing vasculitis in medium- or small-sized arteries or angiographic abnormalities (conventional angiography if magnetic resonance angiography is negative) as a mandatory criterion plus one of the following five: skin involvement, myalgia or muscle tenderness, hypertension, peripheral neuropathy, and renal involvement [4]. In children, various systems are involved in PAN with the skin, the musculoskeletal system, the kidneys, and the gastrointestinal tract most prominently affected; and cardiac, neurologic, and respiratory manifestations occurring less frequently [1].

### Therapeutic advances

Current treatment of PAN in children is based on limited trial data in adults. There are a number of RCTs for treatment of PAN, all relating to adults [40–43]. Conclusions based on RCTs in adults with PAN with important implications for children are: a) treatment of severe PAN requires corticosteroids combined with intravenous cyclophosphamide; b) despite therapy, mortality associated with PAN in adults remains high at 4–22 %; treatment-related toxicity contributes to this; c) adverse events (disease and treatment-related) affect 54–100 % patients; and d) avoidance of cyclophosphamide in children is desirable if alternatives exist since complications associated with cyclophosphamide include infertility and malignancy [40–43]. In line with this, in the largest published cohort of paediatric PAN patients, cyclophosphamide was used in combination with steroids as first line therapy in 83 % of the cases albeit with a number of significant adverse events such as neutropenia and sepsis noted [1]. Based on the fact that MMF appears to be an alternative to cyclophosphamide for induction of remission in other systemic vasculitides and autoimmune diseases with low toxicity profile, the MYPAN study (Mycophenolate mofetil for childhood PAN) was set up to explore whether MMF is non-inferior to cyclophosphamide, and with comparable (or less) short medium term safety in children with PAN [25, 44]. Given that a definitive trial analysed by frequentist statistical methods would require 513 patients per arm and would take well over 30 years to recruit [44], the MYPAN study adopts a Bayesian approach, first characterising expert prior opinion about the 6-month remission rate on CYC and the relative benefit of MMF as probability distributions before the trial begins, and then updating these distributions using Bayes theorem once data become available [44]. Using this rare disease trial design we hope to deliver the first clinical trial in childhood PAN.

Use of biologic agents, including anti TNF-α, and rituximab is also described for children with systemic PAN, particularly those not responding to standard therapy or because of concern regarding cumulative cyclophosphamide toxicity [5]. These therapies have not been formally assessed in RCTs in either children or adults with PAN, however.

## Takayasu arteritis

Takayasu arteritis (TA) is the only large vessel vasculitis (LVV) referred to in the current paediatric classification, affecting the aorta and its major branches [45]. TA has a worldwide distribution, with a reported incidence of 1.2–2.6/million per year in Caucasians, and a 100-fold higher incidence in East Asians [45, 46]. Although the disease rarely affects children, it does occur even in infants [2]. The clinical diagnosis of TA is usually challenging [2, 47, 48]. An initial florid inflammatory vasculitic phase is followed by a later fibrotic/stenotic phase of the illness [2, 47, 48]. It is estimated that one-third of children present within this late fibrotic/stenotic phase of the disease [2, 47, 48]. It is a misconception that this is in some way an “inactive”, or “burnt-out” stage of the disease, since progressive stenotic disease may be the consequence of persistent but low-level large vessel vasculitic disease activity, but without evidence of conventional laboratory markers of systemic inflammation such as elevated C reactive protein, or increased erythrocyte sedimentation rate [2, 47, 48]. Diagnostic delay in children is unfortunately common and almost certainly contributes to worse outcomes [2, 47, 48].

Although hypertension and/or its sequelae is the most common form of presentation in both children and adults, the overall clinical spectrum at presentation of children with TA may differ from that in adults [2, 47, 48]. The most frequent presentation in children is with arterial hypertension (82 %) followed by headaches (31 %), fever (29 %), dyspnoea (23 %), and weight loss (22 %) [2, 47, 48]. Musculoskeletal symptoms affect approximately 14–65 % of children with TA [2, 47, 48]. In contrast, adults rarely report arthritis or arthralgia. Bruits (48 %) and claudication (27 %) are more commonly reported in adults with TA [2, 47, 48]. Ocular manifestations are also rare in children [2, 47, 48]. A detailed overview of imaging in TA is beyond the scope of this review, and we refer the reader elsewhere for this [2].

### Therapeutic advances in TA

There have been no randomised controlled trials to guide treatment, and few evidence-based therapeutic advances. Biologic therapies are increasingly used in children, particularly anti-TNFα [5], and anecdotally are reported to be efficacious. A major therapeutic challenge, however, is that there remains significant diagnostic delay for TA in children. This leads to significant and sometimes irreversible damage in the pre-diagnostic phase of the illness. At the time of writing, a major clinical trial of tocilizumab (a monoclonal antibody against the interleukin 6 receptor) for the treatment of giant cell arteritis in adults is ongoing [49], and hopefully will report later in 2016. It is debatable, however, what relevance this might have for children with TA.

SHARE guidelines for the treatment of TA in the paediatric population will be formally published soon. The general therapeutic approach is that of induction of remission (high dose corticosteroid combined with another immunosuppressant), followed by maintenance of remission therapy (lower dose corticosteroid combined with a maintenance immunosuppressive agent, usually methotrexate), or institution of second line therapy for failed induction [2]. Corticosteroids are the mainstay of first-line treatment for TA [2]. In addition, methotrexate, azathioprine, MMF, and cyclophosphamide have been used in children as first or second line agents [2, 47, 48]. Ozen, et al. described 6 children with TA, and treatment with steroid and cyclophosphamide induction followed by MTX was suggested as effective and safe for childhood TA [50]. Anti-TNF therapy may be beneficial [2, 51]. Promising results have also been reported with anti-IL6 therapy (tocilizumab) for adults with TA, and in some cases children [52, 53]. Surgical intervention is frequently required to alleviate end-organ ischemia and hypertension resulting from vascular stenosis, although it is preferable to control the vasculitic process before performing revascularisation procedures or other vascular surgery, if possible, since outcomes are worse if these are undertaken when the disease is still active [2, 47, 48].

The 5 year mortality rate of TA in children has been reported as high as 35 %; we recently reported 27 % mortality from TA in children [2]. Prognosis is dependent upon the extent of arterial involvement and organ damage at presentation; age of patient at disease onset (children under 5 have poorer prognosis); and on the severity of hypertension [2, 47, 48]. It is unlikely that a randomised controlled trial for TA in children will be undertaken in the foreseeable future; extrapolation from adult trials (such as the soon-to-be reported tocilizumab trial) will provide some evidence base, but with all the caveats around using data from adult trials to inform paediatric practice.

## Monogenic vasculitides: DADA2; CANDLE; and SAVI

Three recently described monogenic autoinflammatory conditions with a major vasculitic component are worthy of description in brief, since they highlight the concept that understanding the molecular pathogenesis may lead to more targeted therapy [54–57].

### DADA2

Deficiency of adenosine deaminase type 2 (DADA2) is an autosomal recessive disease resembling polyarteritis nodosa, caused by homozygous or compound heterozygous mutations in the *CECR1* gene [54, 55]. The cardinal clinical features include livedo racemosa, neurological involvement including propensity to lacunar (small vessel) stroke, vasculitic peripheral neuropathy, digital ischaemia and cutaneous ulceration, systemic inflammation, and other end organ damage [54, 55, 58, 59]. There is an emerging view that anti TNF alpha is particularly efficacious for this form of monogenic vasculitis [59]; this may be due to the fact that the extracellular enzyme ADA2 functions as an important regulator of immune development. Patients with DADA2 demonstrate skewed macrophage development towards the M1 pro-inflammatory phenotype as opposed to the M2 anti-inflammatory phenotype [54, 55]. M1 macrophages are known to produce TNF alpha, which could explain why this therapeutic approach seems particularly effective in DADA2 [54, 55]. Allogeneic haematopoietic stem cell transplantation has been reported to be successful in a few patients [60]; gene therapy may be an option for the future [59].

### CANDLE and SAVI

**Fig. 2:**
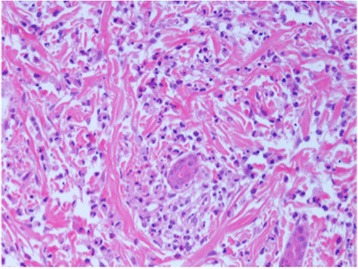
High power view of skin biopsy taken from an 8 week old infant with CANDLE syndrome caused by digenic mutation in PSMB4 and PSMB9. There is a florid leukocytoclastic cutaneous vasculitis with karyorrhectic debris. In most areas distinct vessels could not be identified due to the destructive vasculitic process

CANDLE syndrome (Chronic Atypical Neutrophilic Dermatosis with Lipodystrophy and Elevated temperature) is a recessive disease caused by homozygous, compound heterozygous or digenic mutations in the proteasome pathway, and is classified as a proteasome-associated autoinflammatory syndrome (PRAAS) [57, 61]. Mutations in PSMB8, PSMB4, PSMB9, PSMA3, and proteasome maturation protein (POMP) are described [57, 61]. In the early stages, the neutrophilic dermatosis may display the histological features of neutrophilic/leukocytoclastic vasculitis (Fig. 2). CANDLE is associated with dysregulated type I interferon production, therefore targeting this pathway with Janus kinase (JAK) inhibitors may be a promising novel therapeutic approach [57, 61]. Early clinical trials of baricitinib, an oral JAK1 and JAK2 inhibitor, are ongoing.

**Fig. 3:**
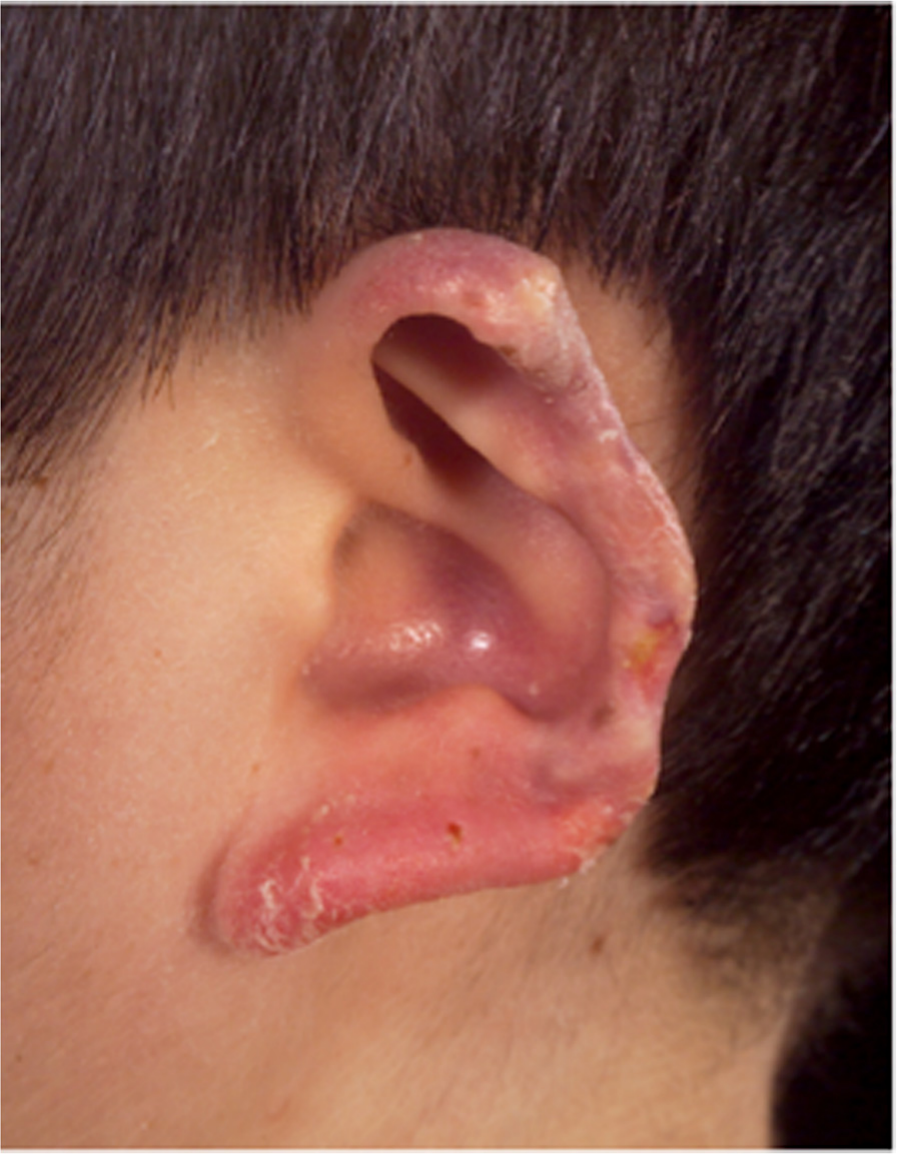
Chronic scarring of the pinna in a 12-year old boy with SAVI

Stimulator of interferon genes (STING)-associated vasculitis of infancy (SAVI) arises from sporadic/dominant mutation in the *TMEM173* gene and presents early in life with a vasculitic rash affecting the cheeks, nose, and peripheries (Fig. 3) with chronic ulceration; and progressive interstitial pulmonary fibrosis and associated pulmonary hypertension [56, 62]. Standard vasculitis therapies are ineffective. Cutaneous vasculitis and deteriorating lung function usually continue relentlessly throughout childhood, with development of pulmonary hypertension and lung fibrosis, often with fatal outcome [56, 62]. SAVI is considered part of the growing group of Mendelian disorders recognised as “interferonopathies” which include Aicardi–Goutières syndrome (AGS), and the aforementioned CANDLE syndrome [63]. Whilst there are virtually no published data currently available, anecdotal reports again suggest that early treatment targeting the interferon pathway (e.g. with JAK inhibitors) currently offers the best hope for survival.

### Concluding remarks

Considerable therapeutic advances for the treatment of vasculitis of the young have been made in the past 10 years, including the development of outcome measures that facilitate clinical trial design. Notably, these include: a recognition that some patients with KD require corticosteroids as primary treatment combined with IVIG; implementation of rare disease trial design for PAN to deliver the first randomised controlled trial for children; first clinical trials involving children for ANCA vasculitis (MYCYC and PEPRS); and identification of monogenic forms of vasculitis that provide an understanding of pathogenesis facilitating more targeted treatment [31, 44, 54–57]. Priorities for future research have been identified including a need for robust randomised controlled trials for HSPN; therapeutic trials for TA; and an over-arching need for trials examining new agents that facilitate corticosteroid sparing, of particular importance in the paediatric population since glucocorticoid toxicity is a major concern.
